# Printing and Engraving

**Published:** 1888-07

**Authors:** 


### PRINTING AND ENGRAVING.

The wonderful strides which have been made within the past few years in the reproduction of type forms and engravings, cannot be better explained than by the following statement of the process by means of which the Henry G. Allen Co, reproduce in exact counterpart the entire text, maps and illustrations of that renowned work, the Encyclopedia Britannica which contains forty times more reading matter than the Bible. The following extract explains the operation.

"The process by which this is being done, like many inventions of this kind, is extremely simple, and yet requires such delicacy in handling that it has taken years of careful experimenting to bring it to its present state of perfection.

Gelatine, when prepared by this secret process, has the property of becoming almost as hard as metal when exposed to the sunlight, and remaining soft and soluble when kept in the dark. When in a liquid state it is run out into thin sheets about "type high."

A copy of the latest English edition of the Encyclopædia is cut up and carried to the photographer's room in the engraving establishment. The pages are put in front of large cameras and photographed. The result is a black and white negative—densely black and absolutely translucent—making a fac-simile of the type pages. In the negative the black part of the page—that is the paper—is densely black.

These negatives are next placed in frames over the sheets of gelatine, and exposed to the sunlight. Where the sun shines through the negative the gelatine becomes almost as hard as metal; where the black part of the negative protects the gelatine it remains soft and soluble. The gelatine sheet thus "printed" is taken into a dark room and washed with a brush and ordinary water. The soluble portion disappears, leaving the hardened part—that is, the type—standing up in bold relief.

It is, practically, exactly like a page of type set up and ready for the press.

The illustrations are made in the same way."

It is known to most readers that none of our great New York dailies are printed from the metal type which the compositor uses. The forms are all reproduced in *papier-mache*, then copper faced and fixed upon the cylinders of rotary presses, now exclusively used in large establishments for newspaper work. The process of photo-engraving as practiced by some very extensive establishments was the invention of Mumber so widely known as a spirit-photographer, now deceased.

